# Supplementary material for: Associations between accurate measures of adiposity and fitness, blood proteins, and insulin sensitivity among South Asians and Europeans
Source: medRxiv. 2024 Sep 7:2024.09.06.24313199. Preprint. [Version 1] doi: 10.1101/2024.09.06.24313199 (PMC11398600; doi:10.1101/2024.09.06.24313199)
Supplement: Supplement 1 [file media-1.pdf]

Supplementary Materials

Figures

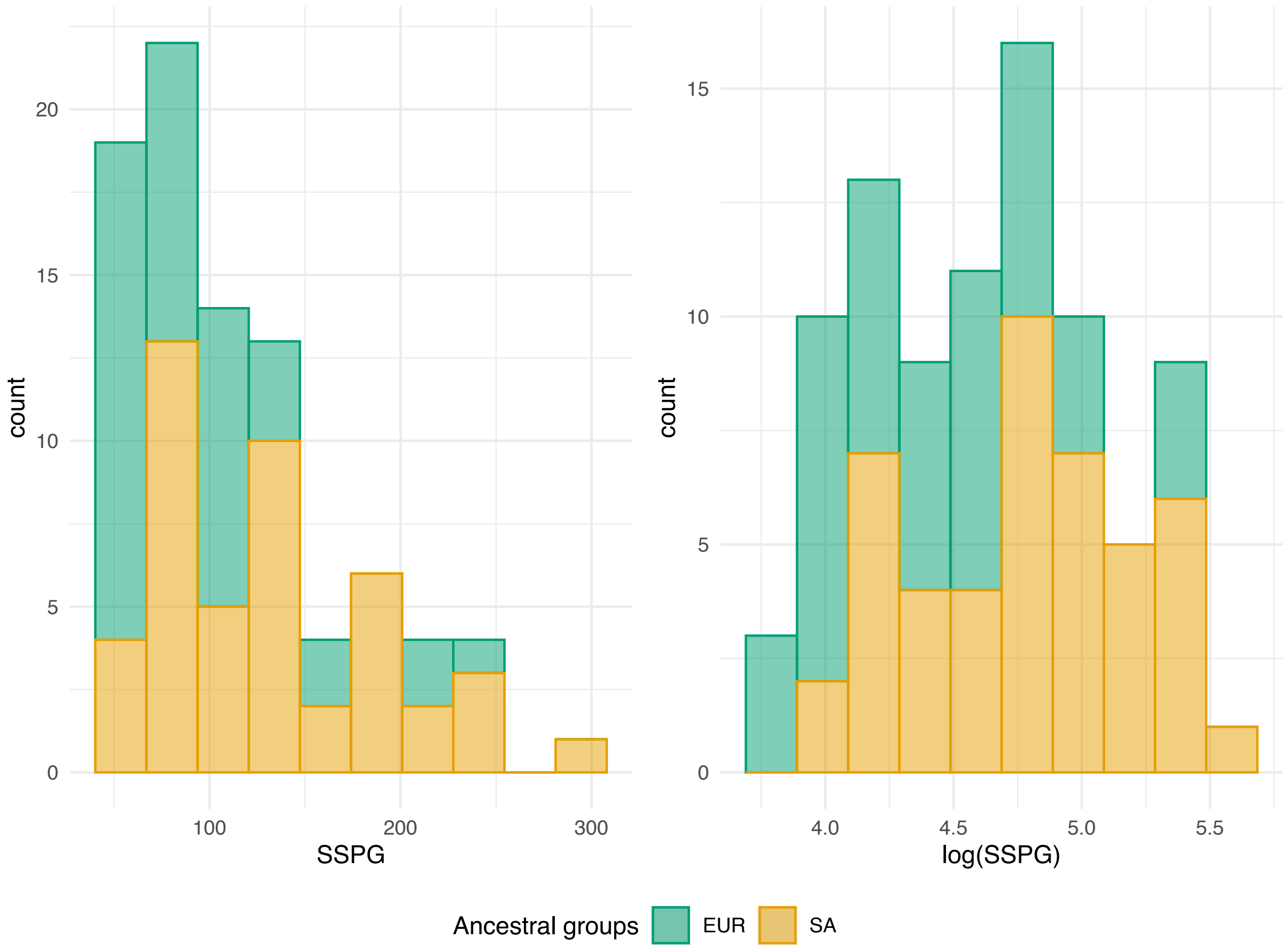

Supplementary Figure 1. Distribution of SSPG and log(SSPG) values. The colors indicate the different ancestral groups: EUR (green) and SA (orange).

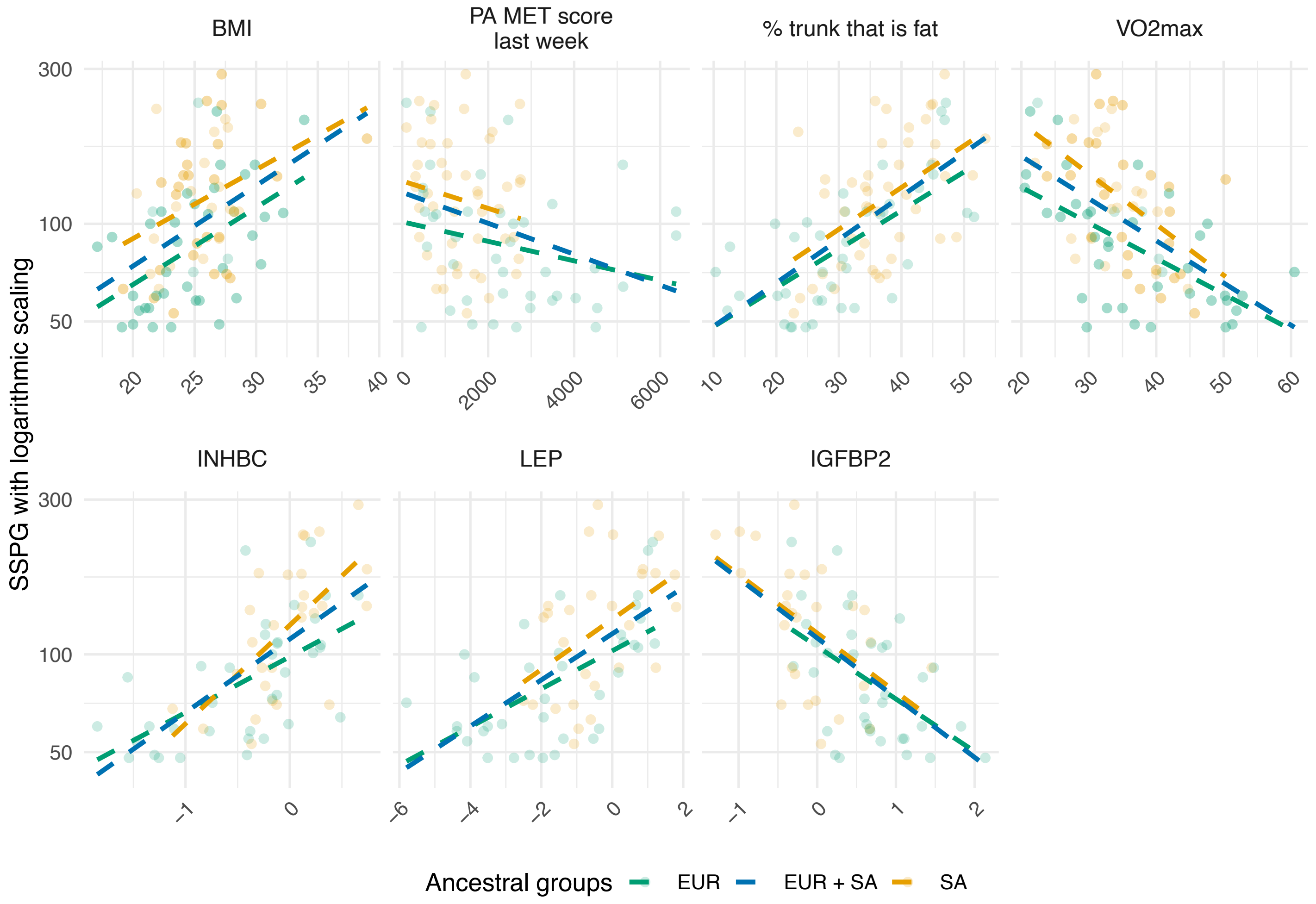

Supplementary Figure 2. Scatter plot between SSPG and candidate predictors for EUR and SA participants. Data points were colored according to ancestral group (green for EUR, orange for SA), with y-axes on logarithmic scale. Dashed lines show linear regression fits, using the same colors as the data points, while the blue dashed lines show the linear regression fit for both ancestry groups combined, without any additional covariates.

Tables

Supplementary Table 1. Proteins and their full names with UniProt IDs

UniProt ID	Protein	Full Name
Q9Y653	ADGRG1	Adhesion G Protein-Coupled Receptor G1
P12104	FABP2	Fatty Acid Binding Protein 2
P15090	FABP4	Fatty Acid Binding Protein 4
P08833	IGFBP1	Insulin Like Growth Factor Binding Protein 1
P18065	IGFBP2	Insulin Like Growth Factor Binding Protein 2
P55103	INHBC	Inhibin Subunit Beta C
P06756	ITGAV	Integrin Subunit Alpha V
P41159	LEP	Leptin
P06858	LPL	Lipoprotein Lipase
Q9BZR6	RTN4R	Reticulon 4 Receptor
Q96PL1	SCGB3A2	Secretoglobin Family 3A Member 2

Supplementary Table 2. Coefficient of ancestry and adjusted R2 values from regression analysis of log(SSPG) with selected groups of variables, adjusted for age, sex and ancestral group

	SAs vs EURs			Adjusted R^2			Residual SE		
**Model**	**Beta**	**SE**	**P**	**Combining ancestral groups**	**SA only**	**EUR only**	**Combining ancestral groups**	**SA only**	**EUR only**
Base model (age, sex)	0.471	0.125	4.10E-04	-0.026	-0.045	0.069	0.489	0.486	0.414
Conventional measures of adiposity and physical activity (BMI and PA MET scores last week)	0.286	0.119	0.019	0.320	0.060	0.407	0.398	0.461	0.330
Gold standard measure of adiposity (% trunk that is fat) and cardiorespiratory fitness (VO_2 max)	0.142	0.116	0.228	0.452	0.357	0.368	0.357	0.381	0.341
Plasma proteins (INHBC, LEP, IGFBP2)	0.069	0.114	0.547	0.546	0.573	0.489	0.325	0.311	0.307

For model with sex, age and ancestral group, adjusted R^2=0.154 and residual SE=0.444. SAs, South Asians; EURs, Europeans; R^2, variance; SE, standard error; BMI, body mass index, PA, physical activity, MET: metabolic equivalent, VO2 max, amount of oxygen utilized during a cardiopulmonary exercise test with maximal effort, INHBC, Inhibin Subunit Beta C; LEP, Leptin; IGFBP2, Insulin Like Growth Factor Binding Protein 2
